# Route retracing: way pointing and multiple vector memories in trail-following ants

**DOI:** 10.1242/jeb.246695

**Published:** 2024-01-25

**Authors:** Cody A. Freas, Marcia L. Spetch

**Affiliations:** ^1^ Department of Psychology, University of Alberta, Edmonton, AB, Canada, T6G 2E9; ^2^ School of Natural Sciences, Macquarie University, Sydney, NSW 2109, Australia

**Keywords:** Celestial compass, Trap-lining, Waypoints, Pheromone trails, *Veromessor pergandei* ants, Local vectors

## Abstract

Maintaining positional estimates of goal locations is a fundamental task for navigating animals. Diverse animal groups, including both vertebrates and invertebrates, can accomplish this through path integration. During path integration, navigators integrate movement changes, tracking both distance and direction, to generate a spatial estimate of their start location, or global vector, allowing efficient direct return travel without retracing the outbound route. In ants, path integration is accomplished through the coupling of pedometer and celestial compass estimates. Within path integration, it has been theorized navigators may use multiple vector memories for way pointing. However, in many instances, these navigators may instead be homing via view alignment. Here, we present evidence that trail-following ants can attend to segments of their global vector to retrace their non-straight pheromone trails, without the confound of familiar views. *Veromessor pergandei* foragers navigate to directionally distinct intermediate sites via path integration by orienting along separate legs of their inbound route at unfamiliar locations, indicating these changes are not triggered by familiar external cues, but by vector state. These findings contrast with path integration as a singular memory estimate in ants and underscore the system's ability to way point to intermediate goals along the inbound route via multiple vector memories, akin to trapline foraging in bees visiting multiple flower patches. We discuss how reliance on non-straight pheromone-marked trails may support attending to separate vectors to remain on the pheromone rather than attempting straight-line shortcuts back to the nest.

## Teaser

**Summary:** Trail-following ant foragers navigate to distinct intermediate sites via path integration, underscoring the ability ignore their global vector to way point via multiple vector segment memories.

## INTRODUCTION

Efficient homing to goals is a critical recurring challenge for navigating animals, often necessitating the navigator track its position in relation to these sites, even in habitats that are visually featureless. Insect navigators possess several strategies to efficiently orient to compass headings and reach goals (Heinze et al., 2018; Wehner, 2020; Freas and Cheng, 2022). One widely documented solution is path integration (PI), where the navigator maintains an updating positional estimate relative to its origin by measuring distance and directional movement along the route, integrating these into a global vector (Wehner and Srinivasan, 1981; Wittlinger et al., 2006).

Many invertebrate navigators are well documented to attend to a PI-derived vector to return directly, along a straight-line route to their nest/burrow rather than re-tracing their winding outbound paths (Hymenoptera: Cheng, 2006, Freas and Spetch, 2023a; Collett and Collett, 2000, Patel et al., 2022; Srinivasan et al., 1997; Wehner and Srinivasan, 2003; beetles: Dacke et al., 2020; flies: Kim and Dickinson, 2017; cockroaches: Durier and Rivault, 1999; crabs: Zeil and Layne, 2002; spiders: Ortega-Escobar, 2002; Ortega-Escobar and Ruiz, 2010; Sergi et al., 2021).

In ants, PI is often modelled as a single estimate, integrating pedometer and celestial compass information to produce a global vector that directs returning individuals back to the nest, with a distinct lack of the ability to home to intermediate sites en route. In visually cluttered environments, as individuals become experienced with the landmarks along their established foraging route, they develop stereotypical paths detouring around any obstacles (Wystrach et al., 2011, 2020; Mangan and Webb, 2012; Freas and Cheng, 2019). These detours often involve periods of turning away from the global vector before reorienting back to the nest direction (Collett et al., 1992; Bisch-Knaden and Wehner, 2001; Wystrach et al., 2011; 2020; Mangan and Webb, 2012).

One theoretical mechanism underlying detouring, introduced by Collett et al. (1993, 1998), is that ant (and honeybee) navigators can suppress their global vector and retrieve a ‘local vector memory’, a directional and distance estimate of a portion of the full route (Collett et al., 1993; 1998; Srinivasan et al., 1997; Menzel et al., 1998; Collett and Collett, 2009, 2015). Local vectors are defined as PI-derived vector memories independent of both the global vector and current vector state, triggered in combination with familiar panoramic views at the site (Collett et al., 1998, 2002; Bisch-Knaden and Wehner, 2001; Collett and Collett, 2015). Importantly, and perhaps controversially given our current understanding of view alignment, these familiar panorama views trigger the retrieval of local-vector memories rather than directly provide directional information. Yet, this presence of the familiar panorama results in many instances in which examples of foragers using a local vector might also be explained through view alignment alone or orientation via motor routine (Baddeley et al., 2011, 2012; Lent et al., 2013; Hoinville and Wehner, 2018; Webb, 2019; Wolf, 2011). This propensity for navigating ants to engage in view matching has made untangling multiple vector memories from view guidance difficult, leading to them being absent in recent PI models (Heinze et al., 2018; Hoinville and Wehner, 2018; Stone et al., 2017). However, the architecture of the central complex, the brain region associated with the path integrator, is well suited for multiple vector memory use in way-pointing and multi-destination navigational behaviours (Le Moël et al., 2019).

Another multi-destination behaviour is trapline foraging, observed in honeybees and bumblebees. Here, individuals visit multiple flower patches before returning to the hive, developing efficient multi-destination routes (Ohashi et al., 2006; Lihoreau et al., 2012; Buatois and Lihoreau, 2016). Modelling suggests that these routes can arise within the PI system via the navigator switching between multiple vector memories of each flower patch, stored during previous trips, to determine the goal site with the shortest distance to the navigator's current location (Le Moël et al., 2019).

Recent work in trail-following ants provides evidence that multiple vector memories allow for similar multi-destination routes in ants, with foragers travelling to intermediate points along the route, rather than the global vector, when their pheromone trail detours around obstructions (Freas et al., 2020). Compelling evidence of the use of multiple vector memories to home to waypoints comes from the desert harvester ant, *Veromessor pergandei*. Inbound *V. pergandei* foragers attend to only a portion (‘fan vector’) of their global vector while not in contact with the trail pheromone to first return to the pheromone trail rather than directly returning to the nest (Freas et al., 2020).

Multiple vector use is underpinned by *V. pergandei*’s foraging ecology, a column-and-fan foraging structure. Individuals initially leave the nest navigating socially along a pheromone column (up to ∼40 m long) before the pheromone ends (column head) and foragers fan out (1–3 m) alone, off the pheromone, to collect food (Plowes et al., 2013, 2014). Throughout this journey, in both the column and the fan, foragers rely on an accumulating PI-derived vector to navigate and do not engage in view alignment (Freas et al., 2019a,b; Freas and Spetch, 2021). After collecting food, individuals navigate via only a portion of their global vector to first reach the pheromone column before reorienting toward the nest (Freas et al., 2020). The switch from the fan to the global vector, corresponding with being ‘on’ or ‘off’ the pheromone, is mediated by the pheromone cue, with exposure leading to the re-emergence of the vector segment accumulated within the column and an orientation change to the global vector direction. In this way, arriving at the intermediate goal, the pheromone, triggers a goal shift from the column head to the nest.

These findings point to the pheromone column functioning as a critical waypoint along the nest-ward route with an olfactory trigger. Yet, the underlying mechanism within the PI system is currently unknown as it is unclear whether this behaviour represents the capacity of multiple vector segment memories or whether, fitting with local vector theory, portions of the global vector can be neurally suppressed to produce waypoint homing [Le Moël et al.'s (2019) neural modelling supports multiple vector memories over partial suppression]. Interestingly, *V. pergandei* (see also *Formica obscuripes*; Freas and Spetch*,* 2023b) also shows evidence of attending to vector segments within non-straight columns, feats which must have a separate triggering mechanism from pheromone presence observed in the fan. When inbound foragers are collected from non-straight-line pheromone columns and tested at unfamiliar locations, forager paths do not align with the global vector but only to the current inbound route segment (figure 4E of Freas et al., 2020; figure 2C of Freas and Spetch, 2023b). However, untangling orientation to the global vector versus a vector waypoint memory can be difficult because of the small directional differences observed during previous testing (15 and 40 deg, respectively).

Here, we explored multi-vector way pointing while on the pheromone trail by experimentally creating two-legged and three-legged column routes to and from food sites, increasing the directional differences between the overall global vector and its underlying route segments and then testing foragers from these routes at a distant, unfamiliar site (Fig. S1). We found that foragers navigating along non-straight routes did not home via their global vector and instead navigated via distinct segments of their PI (vector memory segments), allowing them to directionally retrace separate legs of their inbound route. As this behaviour occurred at a distant site, multi-destination vector homing was not triggered by familiar external cues, but more likely by the navigator's PI state.

## MATERIALS AND METHODS

### Study species

For the current study, we tested individuals from three *Veromessor pergandei* (Mayr, 1886) nests all located within the Verrado Temporary Trails in Buckeye, AZ, USA (33°19′43.10′′N, 112°01′02.60′′W), with nests spread over a 152.0 m area (nest 1 to nest 2: 152 m; nest 1 to nest 3: 83 m; and nest 2 to nest 3: 87 m). Testing with nest 1 was completed during October to November 2019 while testing with nest 2 and nest 3 was conducted during October to November 2021 and March to April of 2022, respectively. Across conditions, only foragers holding a visible piece of food, cookie pieces provided at the designated feeding site/column head, were tested to ensure homeward motivation, and testing began 10 min after the first foragers in the column reached the feeder site. After testing, foragers were marked as tested and returned to the nest, where they continued to forage over the ensuing days.

There are no governmental regulations guiding the research of invertebrates in AZ, USA. Manipulations were non-invasive and tested individuals were returned to the nest.

### Two-leg column

**Fig. 1.**
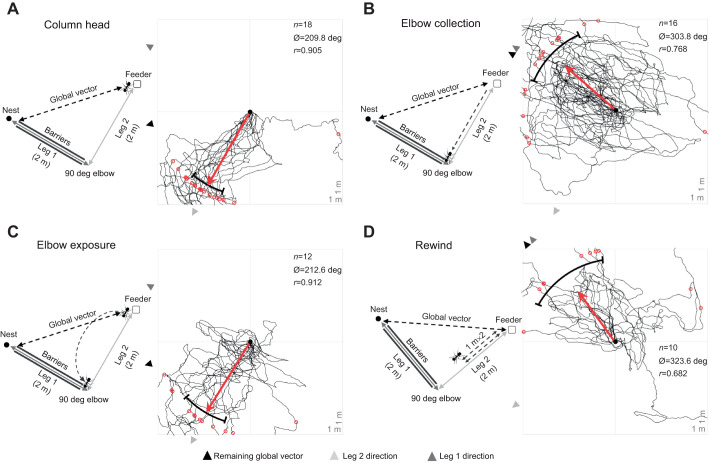
**Experimental layout and forager paths of two-leg column conditions.** (A) Column head, (B) elbow collection, (C) elbow exposure and (D) rewind conditions. Forager paths extend until grid exit, while statistics indicate heading directions as foragers first crossed 1 m from release, marked on each path by a red circle. Black arrowheads: foragers' remaining path integration (PI)-derived global vector; light grey arrowheads: leg 2 compass direction; dark grey arrowheads: leg 1 compass direction; black bars: 95% confidence interval (CI) of headings. Red arrow depicts the mean vector direction and length. *n*, number of individuals; Ø, mean vector; *r*, mean vector length.

After observing the natural column direction on the previous day, we erected a barrier to force foragers to initially leave the nest in a direction with a 45 deg discrepancy from their desired foraging direction (food site). Before morning activity commenced at the nest 1 entrance, 10.0 cm high barriers were placed on the ground around the nest, extending out for 2.0 m and creating a 30.0 cm wide channel for the foraging column to form, travelling away from the nest entrance (Fig. 1A; Fig. S1). This initial segment of the route (nest entrance to the 90 deg elbow, 2.0 m) was designated as leg 1 of the route. This channel was left open to allow the foraging column to shift back to the nest's desired foraging location, leading to a 90 deg counter-clockwise elbow from leg 1 to leg 2. Leg 2 extended 2.0 m (with a ∼50 cm width at leg 2's midpoint). At the end of leg 2, we placed a food site by spreading crushed cookie pieces on the ground in a ∼15 cm diameter area, creating a two-legged column (Fig. 1A). The foraging column extended along each route segment, with depositing behaviour observed along both legs (column formation along both legs took ∼30 min). The column ceased extending once it reached this large amount of food, with no evidence of foragers moving beyond this point. After collecting food, inbound foragers were observed to retrace this two-legged route, rather than leaving the pheromone column along their global-vector direction to return to the nest directly.

Foragers were initially randomly assigned to one of three separate conditions (see below), collected (as they picked up a cookie piece) and displaced to a distant unfamiliar location (>100 m from all nests) that was flat and devoid of vegetation. At this site, a 2 m×2 m grid (1 m squares) was erected using metal pegs and string. Foragers were released at the centre of this grid and their paths were recorded until they left the grid, using an HD GoPro camera (30 frames s^−1^ and 3840×2160 pixel image size) which was positioned 1.5 m above the release point facing straight down. In all conditions using this 2 m×2 m grid, we used these videos to calculate each forager's heading as it first crossed 1 m from its release point. In the first condition, foragers (*n*=18) were collected just as they grabbed a cookie piece from the column head/food site (column head condition; Fig. 1A)*.* In the second condition, foragers (*n*=16) were allowed to travel, with food, back along leg 2 to the 90 deg elbow (elbow collection condition; Fig. 1B) and were collected in the last 10 cm of leg 2 just as they reached the barrier that defined the start of leg 1. In the third condition, foragers (*n*=18) were collected from the column head and released at the 90 deg elbow for 20 s (elbow exposure condition; Fig. 1C), identical to column exposure conditions with fan foragers in Freas et al. (2020). After this exposure period, foragers were re-collected and displaced to the distant site.

These three conditions were completed over two consecutive days while the column structure and direction remained stable. On the morning of the third day, the column was shifted 20 deg counter-clockwise. We re-erected the barriers to create the same two-legged column with a 90 deg counter-clockwise elbow and conducted a final condition (rewind condition; Fig. 1D) where foragers (*n*=10) were ‘rewound’ halfway along leg 2. Rewinding along a route allowed us to update the navigator's path integrator state (to the 90 deg elbow) without exposing the individual to the external cues at the elbow, thereby excluding these cues as triggers of an orientation shift. Foragers with food were allowed to travel halfway back (1 m) along leg 2, where they were collected and released back at the column head to repeat the first 1 m of leg 2 again. After travelling 1 m, these individuals were again collected and tested distantly.

### Two-leg column – full paths

#### Nest 1

**Fig. 2.**
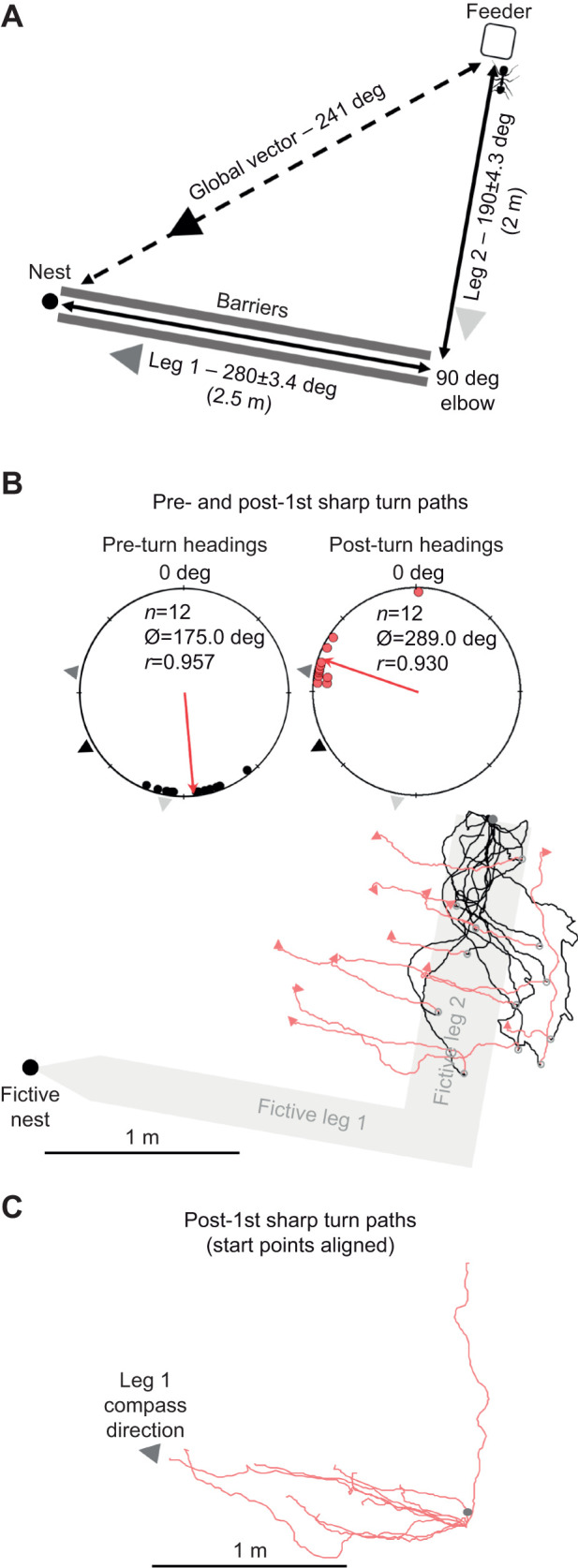
**Experimental layout and forager paths in the nest 1 two-leg column full path condition (distant site).** The fictive column (leg 1 and leg 2) is provided in grey to illustrate the foragers' path alignment with the inbound route. (A) Foraging column with legs 1 and 2 separated by a 90 deg elbow. All foragers were collected from the column head/food site and displaced distantly. Each leg as well as the global vector are coupled with the inbound compass direction (deg) of that route segment. (B) Twelve forager paths (black) until their first sharp (>60 deg) turn (circle) at the distant site and the paths after this first sharp turn (red) until they conduct a second sharp turn (arrow). After this segment, foragers began to search. (C) Aligning the post-sharp turn start points shows the directional alignment of the foragers’ post-sharp turn with the leg 1 compass direction. Black arrowheads: foragers' remaining PI-derived global vector; light grey arrowheads: leg 2 compass direction; dark grey arrowheads: leg 1 compass direction. *n*, number of individuals; Ø, mean vector; *r*, mean vector length.

As we observed evidence of foragers in the column head and elbow exposure conditions turning away from their global vector and orienting instead only along the leg 2 compass direction towards the 90 deg elbow, we chose to further explore this behaviour by conducting a replicate of the column head condition with a separate set of foragers (*n*=12) while recording the full forager paths beyond 1 m. Around nest 1, the barrier along leg 1 was extended 2.5 m with a 30 cm width, while leg 2 remained at 2 m with the column width measuring 50 cm (Fig. 2A; Fig. S1). This extension was meant to ensure foragers had a larger portion of their vector remaining when they reached the elbow, promoting orientation over search behaviour. Additionally, we expanded the grid at the testing site to a 5 m×5 m grid (1 m squares) and again collected foragers from the column head/feeder, just as they grabbed a food piece, and released them at the distant site. Forager paths were again recorded with an overhead camera for 5 min.

When collecting path data, typically the end of navigation via PI and the onset of search is defined as the first sharp turn (a change in heading direction of >50 deg), with the forager continuing in the new direction for at least 20 cm (Schwarz et al., 2017; Freas et al., 2020, 2021). As these new, post-turn heading directions are typically non-straight and uniform in direction, denoting the onset of the systematic search spiral, they are often excluded from analysis. For this experiment, we chose to examine path headings before and after each forager's first sharp turn >60 deg for at least 20 cm (consistent with Freas et al., 2020) to characterize whether foragers began to search after this turn or whether orientation was altered to a new predicted direction (global vector or leg 1 compass direction).

#### Nest 2

**Fig. 3.**
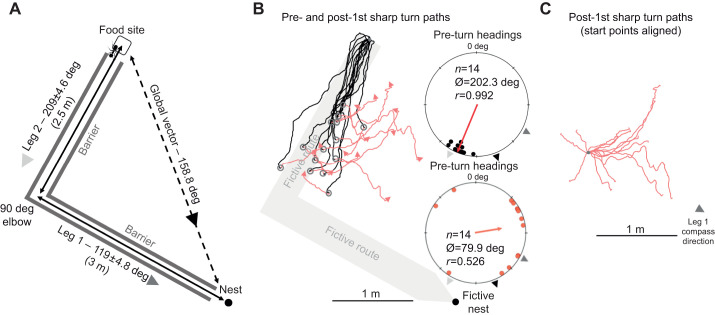
**Experimental layout and forager paths in the nest 2 two-leg column full path condition (distant site).** The fictive column (leg 1 and leg 2) is provided in grey to illustrate foragers' path alignment with the inbound route. (A) Foraging column created with legs 1 and 2 separated by a 90 deg turn. Each leg as well as the global vector are labelled with the inbound compass direction (deg) of that route segment. (B) Twelve forager paths (black) until their first (>60 deg) sharp turn (circle) and post-turn paths (red) until search onset (arrow). (C) Aligning the post-sharp turn start points shows the directional alignment of the foragers’ post-sharp turn with the leg 1 compass direction. Black arrowheads: foragers' remaining PI-derived global vector; light grey arrowheads: leg 2 compass direction; dark grey arrowheads: leg 1 compass direction. *n*, number of individuals; Ø, mean vector; *r*, mean vector length.

After testing with nest 1 foragers, we conducted a full path replicate using a second nest (nest 2). Here, the natural nest heading curved clockwise so that the column passed between two bushes, located 3.0 m from the nest. We established a 90 deg elbow two-legged foraging column (Fig. 3A; Fig. S1) using the same procedure as previous conditions with a 3.0 m long, 50.0 cm wide leg 1, with a 90 deg clockwise elbow and a 2.5 m long, 40.0 cm wide leg 2 (barrier placement and column width were altered because of vegetation/rocks). Foragers (*n*=14) were collected at the column head, just as they grabbed food, and displaced to the distant site, where their inbound paths were recorded identically to previous conditions.

### Three-legged column – full paths

#### Nest 2

**Fig. 4.**
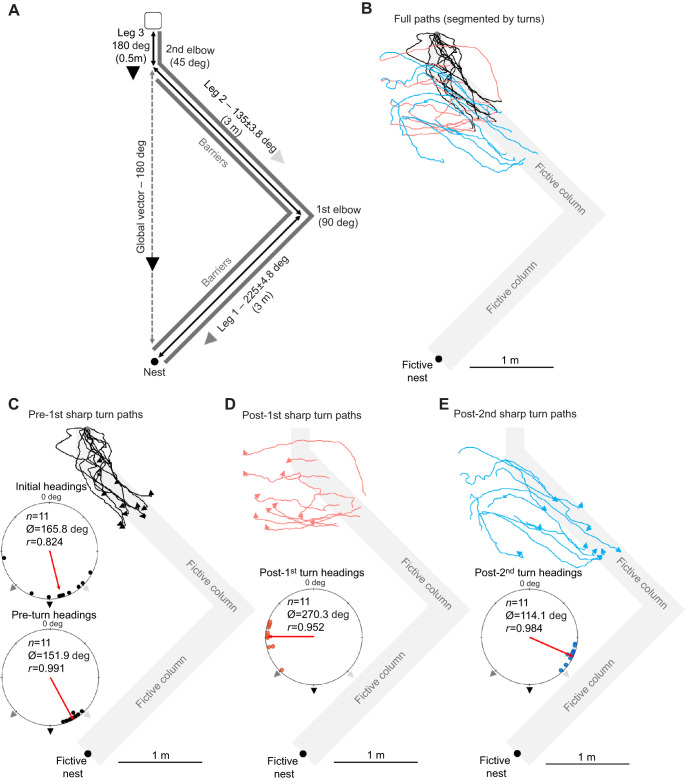
**Experimental layout and forager paths in the three-leg column condition for nest 2.** The fictive column (leg 1, 2 and 3) is provided in grey to illustrate foragers' path alignment with the inbound route. (A) Foraging column with legs 1 and 2 separated by a 90 deg first elbow, while leg 2 and 3 are separated by a 45 deg second elbow. Each leg as well as the global vector are coupled with the inbound compass direction (deg) of that route segment. (B) Full forager paths segmented by segment. (C) Forager paths (black) until their first sharp (>60 deg) turn (arrow). (D) Post-first turn paths (red) of same foragers until a second sharp turn (arrow). (E) The third path segment (blue) after each forager's second sharp turn (headings remained directed). Blue arrows denote the end of the third path due either to a sharp turn or to the end of recording. *n*, number of individuals; Ø, mean vector; *r*, mean vector length.

We next characterized how non-nest orientation (along leg 2) was initiated, by collecting foragers moving along their global vector direction, before their pheromone column shifted away from this direction. Around nest 2, we erected barriers creating a three-legged column (Fig. 4A). Leg 1 of the column extended 3.0 m from the nest entrance (width 50.0 cm) and ended with a 90 deg counter-clockwise elbow (first elbow) and a 3.0 m leg 2 (width 40.0 cm). At the end of leg 2, the arena shifted 45 deg clockwise (second elbow) with a final leg 3 extending 0.5 m, where we designated a food site and the column ended. This arena setup resulted in inbound foragers returning along leg 3 in their global vector before the pheromone column turned away from this direction along leg 2. As in previous tests, foragers (*n*=11) were collected just as they grabbed a food piece and transferred to the distant site, where their paths were recorded using an overhead camera for 5 min. As foragers' inbound paths from the food site along the column initially allowed foragers to orient along a leg (leg 3) which was directionally identical to the forager's global vector for 50.0 cm, at the distant site we collected initial headings at 30.0 cm as well as headings pre and post the first sharp turn, to catalogue any observable initial orientation to this global vector and an orientation change to the leg 2 segment.

#### Nest 3

**Fig. 5.**
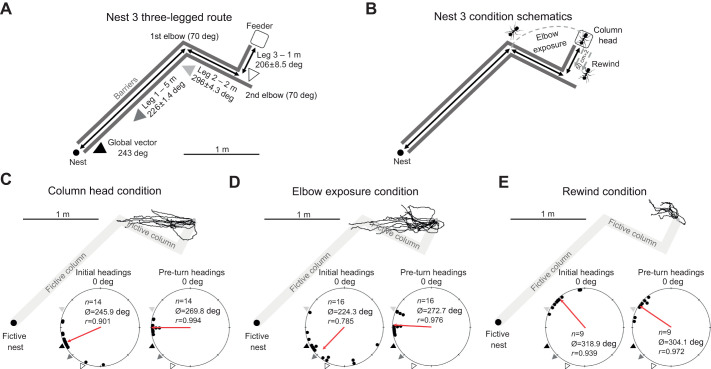
**Experimental layouts and forager paths at the distant site in the three-leg column condition for nest 3.** The fictive column segments of leg 1, leg 2 and leg 3 are illustrated in grey to allow for comparisons of how forager paths align with the inbound column directions and distances. (A) Nest 3 foraging column where legs 1 and 2 were separated by a 70 deg first elbow and leg 2 and 3 were separated by a 90 deg second elbow. Each leg as well as the global vector are coupled with the inbound compass direction (deg) of that route segment. (B) The experimental layout for the three nest 3 conditions. (C) Column head forager paths (black) until their first sharp (>60 deg) turn. (D) Elbow exposure forager paths until their first sharp (>60 deg) turn. (E) Rewind condition forager paths until their first sharp (>60 deg) turn. *n*, number of individuals; Ø, mean vector; *r*, mean vector length.

Testing at nest 3 was conducted to further explore the limits of the foragers' ability to retain multiple vector memories. We erected an arena with three full legs. Leg 1 extended 5.0 m (30.0 cm width) before turning 70 deg clockwise (first elbow) and extending 2 m along leg 2 (30.0 cm width). At the end of leg 2, the arena turned 90 deg counter-clockwise (second elbow) and extended 1 m along leg 3 (30.0 cm width), where we placed a food site to end the foraging column (Fig. 5A). This created a column where inbound foragers after collecting food must first orient 32 deg to the left of their global vector (along leg 3), then turn 54 deg to the right of their global vector to follow along leg 2 of the column. Foragers were collected and randomly assigned to one of three separate conditions (Fig. 5B). In the first (column head) condition, foragers (*n*=14) were collected just as they grabbed a food piece from the column head at the end of leg 3*.* In the second (elbow exposure) condition, foragers (*n*=16) were collected from the column head and released at the 70 deg first elbow between leg 1 and leg 2 for 20 s before being recollected and tested distantly. In the final (rewind) condition, foragers (*n*=9) were rewound along leg 3 to set their vector state as though they were at the second elbow but without being exposed to the cues at this site. Foragers were allowed to travel halfway back (50.0 cm) along leg 3, collected and released back at the column head and then re-collected at the halfway point and tested distantly. Forager paths were recorded at the distant site under the same methodology as previous conditions.

### Statistical analysis

Video analysis was conducted to digitize forager paths using GraphClick. Forager positions from their release point at the distant site grid were recorded by taking their position in 0.2 s increments. In the 1 m heading conditions at nest 1, we calculated the first frame each forager passed a 1 m distance from their release point. Heading directions in each condition were first analysed using Oriana software to determine whether they were directed using Rayleigh tests for circular data (Fisher, 1993, eqns 4.17 and 4.18). If headings were considered to be directed via the Rayleigh test (α=0.05), we further analysed whether the mean direction of observed headings was in a particular predicted direction using the 95% confidence interval (CI) around the mean of forager headings. CIs were calculated through the standard error of the mean heading direction based on the mean vector length (Fisher, 1993, eqn 4.42). When deciding the compass direction of individual legs of the column, we chose the range of angular directions representing the width of the column rather than the specific centre point in order to account for columns forming tightly around inner bends of each elbow. In the three-legged route conditions where we also analysed orientation to the first elbow, we took compass measurements from the food site to the inner edge of this elbow to compare with heading data, because of the column forming at the inner edge of the turn. Between-condition comparisons of mean heading directions were conducted using Watson–Williams *F*-tests (Fisher, 1993, p. 126; Batschelet, 1981, p. 95) while paired comparisons within the paths of individual foragers (i.e. headings pre/post-turn) were analysed using Moore's paired tests (Zar, 1999). When multiple comparisons were made with the same condition, Bonferroni corrections (*a/n*) were used to determine significance level (α=0.025).

## RESULTS

### Two-leg column

Column head forager paths were significantly directed at 1 m (Rayleigh test; *Z=*14.75; *P<*0.001; Fig. 1A) and only the compass direction of leg 2 of the column (210.0 deg±7.1 deg) was within the 95% CI of headings (209.8±11.8 deg), while both the compass directions of the global vector (255 deg) and the leg 1 column (300.0±4.3 deg) were well outside this range, signifying orientation not to the nest but along the (leg 2) route to the fictive position of the 90 deg elbow. This elbow orientation remained consistent for forager headings in the elbow exposure condition, which were also significantly directed (Rayleigh test; *Z=*9.99; *P<*0.001; Fig. 1C) and only the compass direction of leg 2 (210.0±7.1 deg) towards the 90 deg elbow was within the 95% CI of headings (212.6±15.8 deg), while both the global vector (255 deg) and the leg 1 (300.0±4.3 deg) compass directions were well outside this range of headings. Mean heading directions between these conditions (column head versus elbow exposure) did not significantly differ (Watson–Williams *F*-test; *F*_1,28_=0.08; *P=*0.774) suggesting exposure to the external cues present at the elbow did not influence foragers' compass headings.

In contrast, the headings of foragers in the elbow collection condition (303.8±20.2 deg) were significantly directed (Rayleigh test; *Z=*9.44; *P<*0.001; Fig. 1B), yet headings were not directed in the compass direction of leg 2 (210.0±7.1 deg). Instead, leg 1's compass direction at 300.0±4.3 deg, which was also the direction of the foragers’ remaining global vector, was within the 95% CI of headings. Foragers in the rewind condition (323.6±32.1 deg) were similarly directed (Rayleigh test; *Z=*4.64; *P=*0.006; Fig. 1D) in the leg 1 compass direction (320.0±4.3 deg) and remaining global vector compass direction (320.0 deg), but not in the leg 2 compass direction at 230.0±7.1 deg. Mean heading directions between these conditions did not significantly differ between the rewind and elbow collection conditions (Watson–Williams *F*-test; *F*_1,24_=1.07; *P*=0.312), indicating that the change in headings was not triggered by exposure to external cues present at the elbow.

### Two-leg column – full paths

#### Nest 1

Column head-collected foragers exhibited pre-turn headings that were directed (Rayleigh test; *Z=*10.98; *P<*0.001; Fig. 2B) with only the compass direction of the leg 2 column (190.0±7.1 deg) being within the 95% CI of headings (175.0±11.0 deg). Both the global vector (241.3 deg) and leg 1 (280.0±3.4 deg) were well outside this heading range. The mean (±s.e.m.) distance travelled pre-turn (1.14±0.13 m) was well short of the 2 m distance to the 90 deg elbow (57%).

Post-turn headings significantly shifted away from the pre-turn direction (Moore's paired test; *R*_12_=1.80; *P<*0.001). Post-turn headings (289.0±14.1 deg) remained directed (Rayleigh test; *Z=*10.38; *P<*0.001; Fig. 2C) with only leg 1's compass direction (280.0±3.4 deg; Fig. 2C,D) being within the 95% CI of headings. When we individually calculated each forager's remaining global vector compass direction at the turn position, this direction (260.7±5.0 deg) was significantly different from the observed post-turn headings (Moore's paired test; *R*_12_=1.802; *P*<0.001) with headings biased on average 30.6±13.3 deg clockwise of the remaining global vector. The mean (±s.e.m.) distance travelled during this second path segment (0.90±0.13 m) was short of leg 1's 2.5 m distance (36%) before the ants conducted another sharp turn. Beyond this second turn, forager path directions were uniform (Rayleigh test; *P>*0.05), suggesting the onset of search.

#### Nest 2

Column head forager headings were directed (Rayleigh test; *Z=*10.98; *P<*0.001; Fig. 3B) and once again only leg 2 (209.0±4.6 deg) was within the 95% CI of headings (202.3±4.4 deg), while both the compass directions of the global vector (158.8 deg) and leg 1 (119.0±4.8 deg) were well outside this range. Again, foragers did not travel the full 2.5 m leg 2 distance before turning (mean±s.e.m. 1.87±0.12 m), with foragers completing 74.7% of the true distance. After the first turn, foragers' headings significantly shifted away from the pre-turn headings (Moore's paired test; *R*_14_=1.81; *P*<0.001) and these new headings remained directed (Rayleigh test; *Z*=3.87; *P*=0.018; Fig. 3B,C), suggesting foragers had not begun to search. Only the leg 1 compass direction (119.0±4.8 deg) was within the 95% CI of these headings (79.9±39.2 deg), while leg 2 (209.0±4.6 deg) was well outside this range. We again calculated each forager's remaining global vector compass direction from their position at their first sharp turn and this compass direction (139.9±4.4 deg) was significantly different from the observed headings post-turn (Moore's paired test; *R*_14_=1.64; *P*<0.001), with observed mean headings 59.8±35.8 deg to the left of the global vector direction. Post-turn foragers were oriented only to the leg 1 direction of their vector and not to their remaining global vector. After the second >60 deg turn, mean heading directions were uniform and no longer directed (Rayleigh test; *Z*=1.37; *P*=0.257), indicative of the onset of search.

### Three-legged column full paths

#### Nest 2

Column head forager headings at 30 cm were directed (Rayleigh test; *Z*=7.48; *P*<0.001; Fig. 4B) and only the compass direction of the global vector and leg 3 (180.0 deg) was within the 95% CI of these headings (165.8±24.2 deg), while the compass direction of the leg 2 column (135.0±3.8 deg) was just outside this range. The leg 1 direction (225.0±4.8 deg) was well outside the 95% CI of headings. However, the compass direction from release to the first elbow at 149.0 deg, representing a combination of the leg 3 and leg 2 columns, was within the 95% CI of initial headings at 30 cm. After the foragers' first sharp turn, headings remained directed (Rayleigh test; *Z*=10.81; *P*<0.001; Fig. 4C) but had shifted to the left of their initial orientation by 13.9 deg in the direction of leg 2 and the first elbow, though this change was not significant (Moore's paired test; *R*_11_=0.987; *P*>0.05). Interestingly, the global vector compass direction (180.0 deg) was no longer within the 95% CI of headings of foragers at the first sharp turn (151.9±5.2 deg). Leg 2's compass direction (135.0±3.8 deg) also fell outside this range, yet the first elbow direction (149.0 deg) was within the 95% CI, suggesting orientation to a combination of leg 2 and 3 (to the first elbow).

Just as in previous conditions, foragers again underestimated the distance to the first elbow, conducting their first turn well before the true distance (mean±s.e.m. 1.34±0.10 m) at 40.6% of the 3.3 m distance to the elbow. Post-first turn, forager headings significantly shifted away from the pre-turn headings (Moore's paired test; *R*_11_=1.80; *P*<0.001) and headings remained directed (Rayleigh test; *Z*=9.98; *P*<0.001; Fig. 4C). Despite highly directed headings (mean±95% CI 270.3±12.3 deg), all predicted compass directions (global vector: 180 deg; leg 2: 135±3.8 deg; leg 1: 225±4.8 deg) fell outside the 95% CI of headings. These paths extended for 1.11±0.17 m.

Unlike previous conditions, after foragers made their second sharp turn, post-turn headings were still directed (Rayleigh test; *Z*=10.65; *P*<0.001; Fig. 4D), suggesting foragers were not initiating search. Yet, headings during this third path segment were (mean±95% CI 114.1±7.1 deg) counter-clockwise of the global vector direction (180 deg) and neither the leg 2 (135.0±4.6 deg) nor leg 1 (225.0±4.8 deg) column direction was within this range. When we calculated the compass direction of the first elbow from the position of each forager's second turn (123.7±8.2 deg) and compared this with each forager's heading (116.8±9.3 deg), they were not significantly different (Moore's paired test; *R*_11_=0.769; *P*>0.05). In contrast, these headings were significantly different from each forager's remaining global vector at the onset of the third path segment (Moore's paired test; *R*_11_=1.80; *P*<0.001), with headings on average 59.0±7.1 deg to the left of the remaining global vector, suggesting foragers were homing to the first elbow, not the nest. By the end of this path segment, the 5 min recording period expired for 4 of the 11 ants, making further analysis of the remaining 7 ants after this point difficult.

#### Nest 3

In all conditions, foragers exhibited directed initial headings at 30 cm from release (mean±95% CI, column head 245.9±15.4 deg, Rayleigh test; *Z*=11.36; *P*<0.001; elbow exposure 224.3±19.3 deg, Rayleigh test; *Z*=9.85; *P*<0.001; rewind 318.9±15.9 deg, Rayleigh test; *Z*=7.94; *P*<0.001; Fig. 5). Initial mean headings in elbow exposure foragers (collected at the column head but exposed to cues from the first elbow prior to testing) did not significantly differ from those of column head foragers (Watson–Williams *F*-test; *F*_1,28_=2.87; *P*=0.101). In contrast, initial mean headings in the rewind condition were significantly different from those of column head foragers (Watson–Williams *F*-test; *F*_1,21_=45.36; *P*<0.001). In column head foragers, only the global vector (243.0 deg) fell within the 95% CI of initial headings, while in the elbow exposure condition, both the global vector compass direction (243.0 deg) and leg 3 column compass direction (206.0±8.5 deg) were within the 95% CI of headings. In contrast, no predicted compass direction was within the 95% CI of rewind headings, though the compass direction of the leg 2 column direction was only 2.7 deg outside this range at 296.0±4.3 deg.

By their first turn, foragers in all conditions remained oriented (mean±95% CI, column head 269.8±3.8 deg, Rayleigh test; *Z*=13.83; *P*<0.001; elbow exposure 272.7±6.2 deg, Rayleigh test; *Z*=15.24; *P*<0.001; rewind 304.1±10.6 deg, Rayleigh test; *Z*=8.51; *P*<0.001). Just as when comparing initial headings between conditions, there was no significant difference in mean heading direction between the column head and elbow exposure conditions (Watson–Williams *F*-test; *F*_1,28_=0.557; *P*=0.462), suggesting exposure to the cues at the first elbow did not influence forager headings, and rewind foragers, consistent with a PI state placing them at the second elbow, exhibited mean headings that remained significantly different from those of column head foragers (Watson–Williams *F*-test; *F*_1,21_=60.52; *P*<0.001).

In both column head and elbow exposure conditions, headings by the foragers' first turn had shifted significantly from initial heading directions, 23.9 deg (Moore's paired test; *R*_14_=1.865; *P*<0.001) and 48.4 deg (Moore's paired test; *R*_16_=1.847; *P*<0.001) to the right, respectively, while rewind foragers exhibited no significant directional shift (Moore's paired test; *R*_9_=0.763; *P*>0.05). Headings at the first sharp turn in the column head and elbow exposure conditions were no longer directed towards the global vector direction (243.0 deg outside 95% CI) and as we observed during testing with the nest 2 three-legged route, no individual leg compass direction was within the observed 95% CI of headings. However, the compass direction from release to the first elbow at 268.0 deg, representing a combination of legs 2 and 3, was within the 95% CI of headings in both conditions. In rewind foragers, only the compass direction of leg 2 (296.0±4.3 deg) was within the 95% CI of headings, consistent with the rewinding manipulation creating a PI state at the second elbow. Column head foragers travelled 80.8% (mean±s.e.m. 1.81±0.17 m) and elbow exposure foragers travelled 71.8% (1.61±0.16 m) of the (2.2 m) actual distance to the first elbow before turning. Rewind foragers only travelled 41.3% (0.83±0.07 m) of the actual 2 m leg 2 distance before turning.

After foragers' first turn in all three conditions, paths remained directed (mean±95% CI, column head 111.0±45.8 deg, Rayleigh test; *Z*=3.06; *P*=0.044; elbow exposure 105.4±18.7 deg, Rayleigh test; *Z*=10.17; *P*<0.001; rewind 118.3±26.1 deg, Rayleigh test; *Z*=6.41; *P*=0.003); in all of these conditions, however, we observed no evidence of orientation to leg 1 or any other predicted inbound direction. Foragers were instead oriented back in the direction of the release point (88.0 deg in the column head and elbow exposure condition; 116 deg in the rewind condition), suggesting foragers had abandoned inbound orientation and were searching/backtracking to re-enter the pheromone (Fig. S2).

## DISCUSSION

In ants, PI is conceptualized and modelled as a singular updating global estimate of the navigator's start location (nest) in relation to their current position (Wehner, 2020). Theories, such as local vectors, which propose the ability to rely upon independent portions of the global PI to produce detour behaviours (Collett and Collett, 2009, 2015), have largely been abandoned in ants, with these behaviours now believed to be performed via view alignment, motor routines and reinforcement learning (Wolf, 2011; Hoinville and Wehner, 2018; Webb, 2019; Murray et al., 2020; Wystrach et al., 2020; Freas et al., 2022). Yet, our work on fan and column foraging ants shows that these navigators possess the ability to way point along the inbound route before reorienting to the nest using their PI system. These PI-based waypoints indicate a more complex PI system capable of retaining multiple destinations along a route to produce way pointing on the inbound route. Trail-following *V. pergandei* are known to accumulate PI estimates both while in their pheromone-marked column and after they leave this column to search for food alone in a foraging fan (Freas et al., 2019b). Fan foragers first navigate back to the pheromone column before re-orienting to the nest, attending to only the portion of their PI accumulated while off the column, while the rest of the global vector appears absent from their orientation. Successfully reaching this column waypoint is critical for the column portion of the vector to be re-expressed, facilitating the return along the pheromone column to the nest. This behavioural evidence indicates an olfaction-mediated distinction between PI accumulation in the presence and absence of the pheromone, allowing foragers to either: (1) retain a second vector which begins at the column head as they leave the pheromone or (2) retain the ability to home via part of their global PI estimate to reach intermediate sites along the route. Reaching intermediate sites along a route can be beneficial, either when navigators collect from multiple food patches (such as with bees, Ohashi et al., 2006; Lihoreau et al., 2012) or when way pointing back to a pheromone trail (Freas et al., 2020). In the current study, we show this ability also exists within the pheromone column itself, with foragers showing evidence they attend to only a segment of their accumulated global vector within the column, presumably for the purpose of retracing curved routes while maintaining contact with the pheromone during inbound navigation.

Across all conditions, foragers oriented along only part of their global vector estimate towards a waypoint along the inbound pheromone trail (the elbow). Foragers in two-legged routes with distinct segments separated directionally by 90 deg oriented to only the second segment (leg 2), heading first towards the 90 deg elbow instead of the nest (Figs 1, 2 and 3). When inbound foragers were exposed to the cues present at this elbow before testing, while their PI state remained unchanged, forager paths did not update to their global vector or leg 1, instead continuing to orient via only the leg 2 segment (Fig. 1C). When foragers' PI state was updated to the elbow, either through elbow collection (Fig. 1B) or through rewinding (Fig. 1D), headings changed to become directed along leg 1 to the nest (even without exposure to the cues at the elbow). Together, these headings indicate that foragers attend to multiple vector segments instead of their global vector and that the cues (both visual and olfactory) present at the elbow do not trigger this reorientation. Instead, the forager's current PI state underlies the orientation change from attending to only leg 2 to reorienting to leg 1 of the route. Only after running off most of the leg 2 portion of the route did foragers re-orient to leg 1.

Our pre/post-sharp turn path analysis provides further evidence that the orientation switch from leg 2 occurs in the absence of any familiar cues (Figs 2 and 3). Foragers tested distantly oriented along leg 2 and not their global vector before conducting a sharp turn (albeit underestimating the correct distance to the elbow), after which foragers oriented along leg 1 of the inbound route and not their remaining global vector. As this directional change occurred at an unfamiliar location, it is doubtful that terrestrial view-based cues triggered the observed orientation change. Instead, after the forager runs off most of its leg 2 vector segment, it reorients to leg 1.

### Multiple vector memories

PI-based waypoints would be in clear contradiction with PI as a singular memory estimate of the nest and points to the PI system's ability to way point to intermediate goals along the inbound route, via the retention of multiple-destination vector memories. Most current models of PI in insects do not account for way pointing in ants (Heinze et al., 2018; Hoinville and Wehner, 2018; Stone et al., 2017; Webb, 2019). However, modelling work envisaging extensions from the known central complex neurons described in Stone et al. (2017) indicates that the central complex is likely well suited to support routes visiting multiple locations through the selection of multiple vector memories (Le Moël et al., 2019). As such, the current study provides clear evidence in ants that multi-destination routes can be represented within the PI system, similar to the ‘trap-lining’ behaviours observed in bees (Ohashi et al., 2006; Lihoreau et al., 2012). Beyond modelling, behavioural evidence of odometer estimates in Hymenoptera also supports the possibility of multi-destination vector memories. Honeybees have been shown to possess two distinct odometer memories, one to communicate to nest-mates and one personal (Dacke and Srinivasan, 2008), while ants possess two separate distance estimates informed via optic flow and the pedometer (Wolf et al., 2018).

Characterizing how PI based way pointing functions requires untangling how segmenting portions of the outbound route could be articulated in the brain (Le Moël et al., 2019). These ant navigators are likely able to retain vector-based memories of sites along their outbound route, representing intermediate goal locations. Each ant needs to be able to retrieve a memory of the intermediate sites or the nest site independently and should then ignore an intermediate site vector memory after it has ‘arrived’. Interestingly the current findings indicate these memories would need to be formed during the first outbound trip rather than rely on memories of multiple previous trips for retracing to be useful on the inbound route. How foragers determine these elbow sites within the column during their outbound route remains unclear, though they are likely triggered via the large rotations in the overhead celestial compass or the motor routine which occurs at these elbows.

A second unresolved issue concerns the triggering mechanism to switch between vector memories as, unlike trapline foraging in bees, ants do not stop nor are they rewarded at the intermediate sites on route (Le Moël et al., 2019). While separating fan and column vector memories clearly involves the pheromone's olfactory input signifying ‘arrival’ (Freas et al., 2020), vector memories within the column cannot be triggered by the pheromone's presence. Additionally, other aspects of the pheromone column, including deposit intensity, are unlikely to be the trigger, as elbow exposure did not elicit a heading change. Instead, the forager must first run down a portion of its current vector segment and ‘arrive’ at the site (at least according to its PI) before choosing to reorient along another leg's vector (Le Moël et al., 2019). It is likely the pheromone still plays some role in this re-orientation decision as observed by the premature turns when testing occurred in the pheromone's absence. In contrast, foragers observed reorienting while navigating on the column were observed to be highly accurate at re-orienting at the 90 deg elbow. While running off a vector segment, the forager is probably also continuously attending to the pheromone's presence along its chosen route. Prolonged periods of pheromone absence likely increase uncertainty and early abandonment of the current heading, just as we see in foragers from straight columns abandoning their global vector early (Freas et al., 2020; Freas and Spetch, 2021). Therefore, despite the vector segment switch being triggered by the forager's PI, the pheromone's presence/absence still influences how far foragers attend to each segment, likely as a strategic decision to prevent overshooting column shifts along the homeward route.

### Underestimating segments

No forager from nest 1 or nest 2 completed the full leg 2 distance to the elbow before turning (a few foragers from nest 3 travelled the full distance to the first elbow), or the leg 1 distance post-turn. These early turns are consistent with foragers either underestimating their vector segment distance or re-orienting before reaching the goal's distance (the elbow).

One possibility is that the unfamiliar views at the distant testing site contributed to the early abandonment of the PI, which is well documented in solitarily foraging species (Cheung et al., 2012; Schwarz et al., 2017). However, as this species does not orient via view alignment, or show evidence of terrestrial landmark use in any of its navigational behaviours (see backtracking and local displacements; Freas et al., 2019a,b), it remains unclear whether these foragers would attend to any terrestrial surroundings, familiar or unfamiliar. However, this does raise the possibility that other sensory metrics of familiarity, present along the route but absent at the distant site, may have influenced the observed early abandonment of the intermediate vector. It is also possible, though we believe unlikely, that this early turn away from leg 2 is due to error accumulation within the PI system (leaky path integrator; see Collett and Collett, 2009; Müller and Wehner, 1988; Vickerstaff and Di Paolo, 2005; Wehner, 2020). Within the PI system, a portion of the accumulated distance estimate is gradually lost as distances increase, leading individuals to underestimate their homeward vector. However, this loss occurs as the PI system integrates paths over much larger distances than the 2.8 m global vector these ants are accumulating. Additionally, in the current study, nest 2 foragers (three-legged route) re-oriented back towards the first elbow compass direction after their second sharp turn, suggesting that they retained an estimate of that location even after their second turn, making the loss of the remaining leg 2 vector unlikely. Finally, further evidence arguing against this hypothesis is observed in fan foragers, where they exhibited highly accurate fan vector paths to return to the column head (Freas et al., 2020). *Veromessor pergandei* foragers following only their fan-accumulated vector completed their full vector segment distance, beginning their search at the 1.5 m vector distance. All evidence points to the potential for PI loss being negligible at these distances.

Instead, the pheromone's absence during testing is likely a critical factor in the early turns away from the first elbow direction. In *V. pergandei*, the pheromone's presence confirms to foragers they are on the right route but have not overshot the nest, promoting continued vector orientation (Freas and Spetch, 2021). Evidence that the pheromone trail acts as a reassurance cue during navigation has also been observed in other trail-following species (Wetterer et al., 1992; Czaczkes et al., 2011). The pheromone's influence on the PI system is complex, playing a role in maintaining vector orientation, backtracking and the vector segment attendance underlying the fan-and-column foraging structure (Freas et al., 2019a,b, 2020; Freas and Spetch, 2021). The pheromone's presence influences the PI system to the extent that foragers that have no remaining vector, having run off their full global vector, still orient in the vector direction when on the pheromone (Freas and Spetch, 2021), suggesting its presence is profoundly intertwined with how these animals navigate. *Veromessor pergandei* foragers rarely orient to their column vector beyond ∼3 m in the absence of this cue, even when tested after accumulating a column vector extending up to 24.0 m (Plowes et al., 2019; Freas et al., 2019a,b; 2020).

Given the pheromone's importance to how these forager's use their PI system across all these contexts, it appears likely it also underlies the decision to cut short the running of the vector segment. We hypothesize that foragers' early turns are a function of the pheromone's absence during testing and this may act as an indicator that the forager has overshot the turn location. Foragers orient to their vector segment while a significant portion of it remains, even when not in contact with the pheromone as, in natural conditions, the column should continue this compass direction (Freas et al., 2020; Freas and Spetch, 2021). However, as the vector segment is run down and uncertainty increases, the continued absence of the pheromone may be a signal that the forager has gone too far and should turn as most of the remaining column is in a new compass direction. This would make the behaviour very similar to backtracking in this species, with foragers backtracking in the pheromone's absence after completing over half their column vector, but well before reaching the end of their global vector (Freas et al., 2019a). This would also explain the differences we see in accurate distance estimation when testing fan foragers (Freas et al., 2020) compared with the current study, as fan foragers would always be travelling their full fan vector segment distance in the absence of the pheromone, and thus its absence would not be an indication to search early.

### Multi-vector memory limitations

Our observed headings during three-legged route testing imply some interesting constraints to this system. Initial forager headings at nest 2, while oriented along leg 3 and the global vector, could not be untangled from orientation to the first elbow (combining leg 3 and leg 2; Fig. 4). This leaves the possibility that foragers struggled to attend to only leg 3 of the route to reach the second elbow. Meanwhile, with nest 3, forager headings did shift significantly between initial headings and the first sharp turns, yet initial headings were only directed to leg 3 in one of two conditions and could not be untangled from global vector orientation in either, making such a distinction difficult (Fig. 5). By the first turn in both conditions (column head and elbow exposure), foragers were well oriented to the compass direction of the first elbow, once again showing evidence of a single waypoint within the column.

These results may indicate that foragers struggle to orient to multiple waypoints with the PI system only capable of retrieving the vector memories of the first elbow and the nest rather than multiple intermediate sites along the route in sequence. The observed heading changes in nest 3 foragers and the lack of such a change in nest 2 foragers may have been due to the larger directional shift preceding leg 3 (90 deg versus 45 deg) or to leg 3 length differences (1 m versus 0.5 m). These differences suggest the possibility that turn magnitude or segment length along the column plays some role in the separation of vector segments within the navigator's memory or a one waypoint limitation mentioned above.

### Trail following

Research on PI in ants is primarily focused on solitarily foraging desert ants (*Cataglyphis* and *Melophorus*), which are diurnal, ‘thermophilic’ scavengers. Given this ecological niche, attending to a global PI to return along the shortest, straight-line distance to the nest, rather than retracing the outbound search, would likely be advantageous by minimizing the time spent exposed to the heat. When species instead forage as a group during periods where temperatures are moderate, maintaining the chemical connection to the nest via contact with the pheromone may be more advantageous to prevent them becoming lost, leading trail-following foragers to retrace the outbound route on their inward journey rather than leaving the pheromone trail to travel along the global vector directly home. Evidence in *V. pergandei* suggests that, while the pheromone trail lacks polarity, it acts as a critical confirmation cue mediating the expression of multiple PI-based navigation behaviours (Freas et al., 2019a, 2020; Freas and Spetch, 2021). Navigational behaviours often rely upon interactions between directional and non-directional cues. Given the heavy importance of the pheromone cue to this species’ PI system, this motivation to return to and maintain contact with the pheromone likely underlies the differences we observed in how *V. pergandei*’s PI system operates compared with that of solitarily foraging ants. For *V. pergandei*, leaving the pheromone is inhibited, meaning foragers should largely avoid global vector shortcuts if they result in leaving the pheromone. Instead, the PI system should direct movement in a way that keeps individuals in contact with the pheromone during the inbound route, leading the PI system to contain a mechanism to allow non-nest-based orientation to both re-enter the pheromone from the fan and allow foragers to retrace curved outbound routes. At present, it remains unknown whether this ability to retrace routes via the PI system alone exists more broadly across ants.

A final behavioural result that eludes our understanding concerns foragers’ post-turn paths during nest 2 testing (second path segment, Fig. 4D) in the three-legged route. These foragers were highly directed yet not in any predicted leg or vector direction, travelling perpendicular to the global vector and away from any point along the outbound route. We posit two possibilities. First, this may have been an attempt to orient to leg 1 that was biased to the right of the true direction. Alternatively, foragers may have been attempting some form of search for the pheromone trail, which was abandoned after a short distance.

### Conclusions

Inbound *V. pergandei* foragers navigate via distinct waypoints to retrace separate legs of non-straight-line routes inconsistent with following a global vector. Attending to multiple vector memories was tested at an unfamiliar site, meaning use was unlikely to be externally triggered and is more likely integrated into their PI during outbound travel. We also show evidence that this ability may be limited within the pheromone column to one waypoint along the homeward route, with foragers along three-legged routes attending to a combination of route segments, suggesting navigation to the first elbow (and not subsequent turns). This way pointing likely relies on the switch between multiple vector memories encoded at points along the outbound route, functioning to allow these trail-following ants to maintain contact with their pheromone throughout the inbound trip.

## Supplementary Material

10.1242/jexbio.246695_sup1 Supplementary information
