# Supplementary material for: Route retracing: way pointing and multiple vector memories in trail-following ants
Source: J Exp Biol. 2024 Jan 25;227(2):jeb246695. doi: 10.1242/jeb.246695 (PMC10906666; doi:10.1242/jeb.246695)
Supplement: Supplementary information [file jexbio-227-246695-s1.pdf]

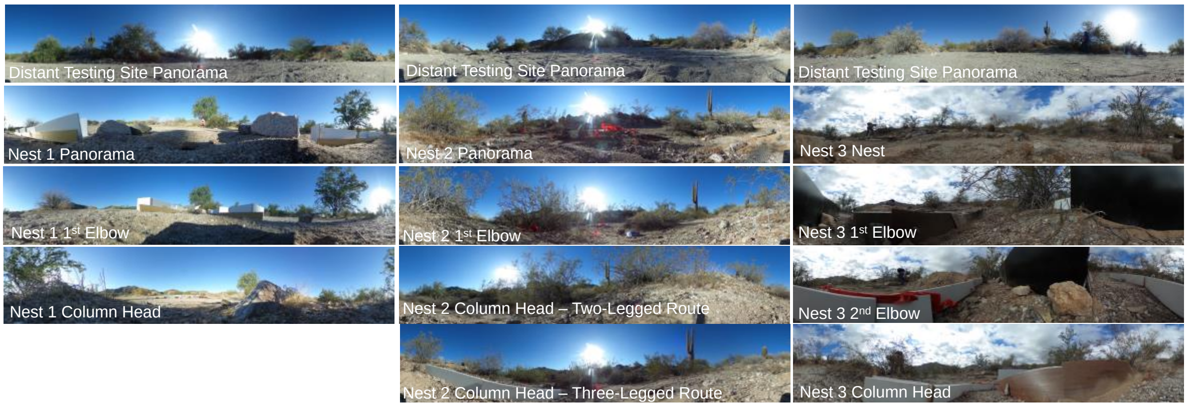

Fig. S1. Panoramic 360° photos of the surrounding visual cues at each Nest and the distant testing site.

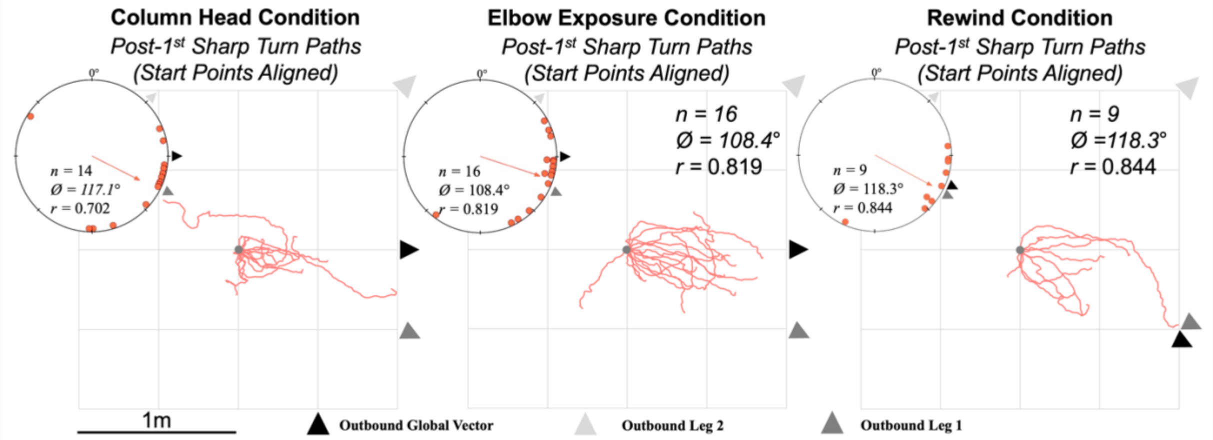

Fig. S2. Forager paths after their first sharp turn at Nest 3. Forager headings in all conditions show clear evidence of directed backtracking and the abandonment of inbound homing. n, number of individuals; Ø, mean vector; r, mean vector length.

Journal of Experimental Biology • Supplementary information
